# A Case of Acute Massive Spinal Subdural Hematoma With a Spot Sign on CT

**DOI:** 10.7759/cureus.97555

**Published:** 2025-11-23

**Authors:** Daigo Aso, Hisaaki Uchikado, Shuji Fukuda, Takehiro Makizono, Nobuhiro Hata

**Affiliations:** 1 Department of Neurosurgery, Saiki Central Hospital, Saiki, JPN; 2 Department of Neurological Surgery, Uchikado Neuro-Spine Clinic, Fukuoka, JPN; 3 Department of Neurosurgery, Fukuoka University Hospital, Fukuoka, JPN; 4 Department of Neurosurgery, Kurume University School of Medicine, Kurume, JPN; 5 Department of Neurosurgery, Oita University Faculty of Medicine, Oita, JPN

**Keywords:** anticoagulant therapy, complication, paraplegia, spinal artery dissection, spinal subdural hematoma, spot sign

## Abstract

Acute massive spinal subdural hematoma (ASSDH) is an extremely rare condition. We report a case of ASSDH in a 74-year-old woman who suddenly developed paraplegia after receiving oral anticoagulant therapy for severe back pain. Reported cases of ASSDH show various clinical presentations, ranging from low back pain to varying degrees of paralysis. In this case, the patient developed acute complete paraplegia. Despite undergoing emergency decompression surgery as soon as possible, the symptoms did not improve. Preoperative angiography revealed a “spot sign” originating from the radiculomedullary artery, suggesting the possibility of arterial dissection. This report mainly describes the preoperative and intraoperative findings of this rare case.

## Introduction

Spontaneous acute spinal subdural hematoma (ASSDH) is an uncommon but potentially devastating condition that can lead to rapid neurological decline. Its incidence is considerably lower than that of intracranial subdural hematoma, and its underlying pathophysiology remains poorly understood. Reported risk factors include idiopathic causes, anticoagulant or antiplatelet therapy, vascular malformations, and sudden increases in intrathoracic pressure [1-3]. Although rare, ASSDH frequently affects the thoracic spine and typically presents with acute back pain, followed by varying degrees of neurological impairment.

Early recognition and timely surgical decompression are crucial for preventing irreversible deficits [4,5]. In intracerebral hemorrhage, the “spot sign” on contrast-enhanced CT is well established as a radiological marker of active bleeding and hematoma expansion [6]. However, to the best of our knowledge, a comparable “spot sign” has not been previously reported in spinal subdural hematomas.

Identifying a spinal “spot sign” may have important clinical implications, as it may help clinicians suspect ongoing hemorrhage and guide urgent surgical decision-making. Here, we describe a rare case of ASSDH exhibiting a CT “spot sign,” supported by intraoperative confirmation of a ruptured radiculomedullary vessel.

## Case presentation

A 74-year-old woman with a history of lung cancer had been receiving long-term warfarin therapy for cancer-related indications. Five hours before arriving at our hospital, she experienced sudden, severe back pain while straining, followed by complete loss of motor and sensory function in both lower limbs. On admission, her vital signs were stable. Neurological examination demonstrated complete paraplegia below the T4 level without sacral sparing, accompanied by urinary retention and loss of anal sphincter tone. Laboratory tests showed a markedly elevated prothrombin time/international normalized ratio (PT-INR) of 4.66.

**Figure 1:**
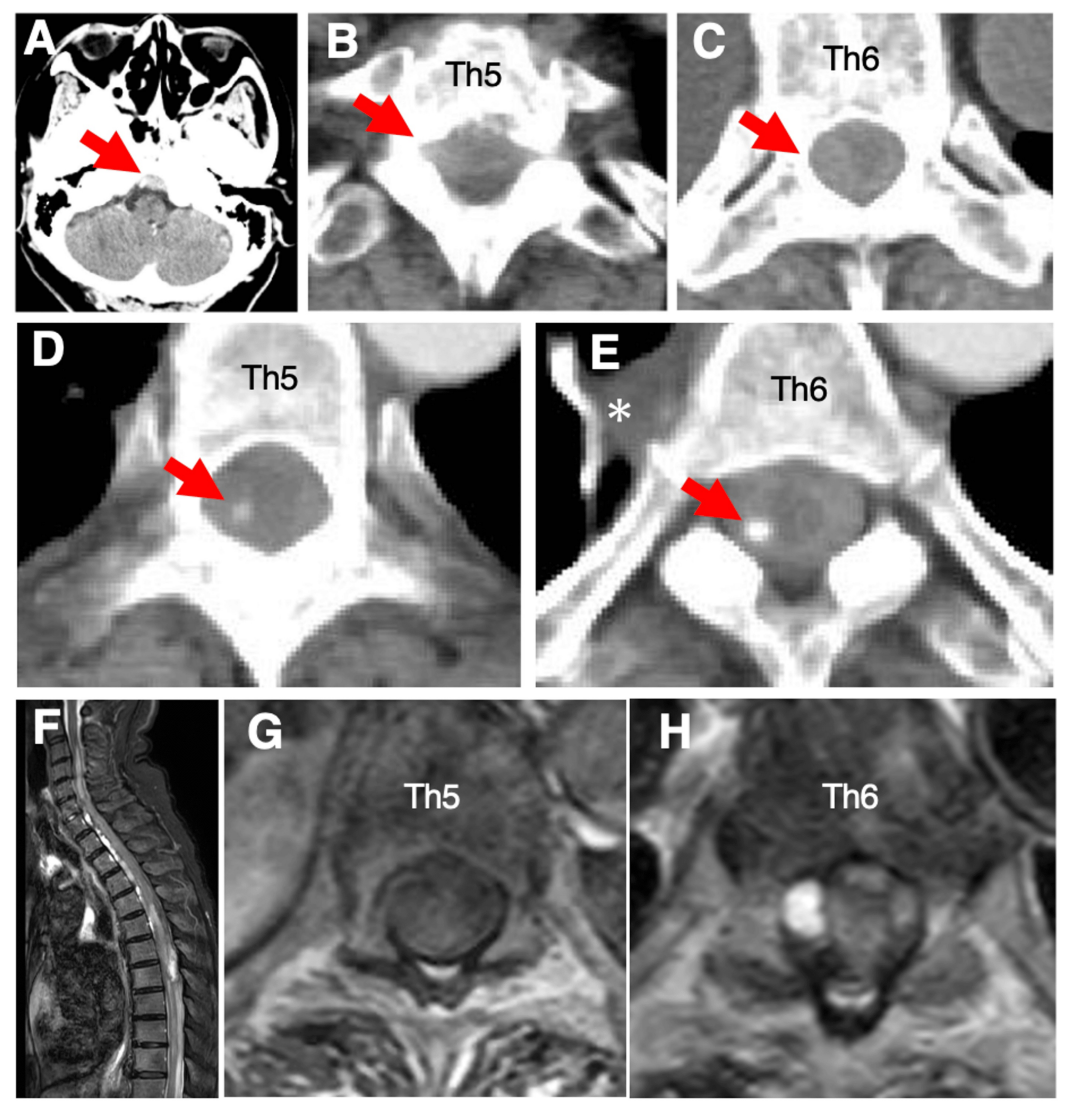
Imaging findings of a case of acute spinal subdural hematoma (A–C) CT images from the previous hospital revealed a hematoma (red arrows) extending from the epidural space of the clivus to the spinal canal of the cervical and thoracic spinal cord (A: head axial; B: Th5 axial; C: Th6 axial). (D, E) Contrast-enhanced CT obtained six hours after symptom onset showed a spot sign at the Th5–6 level (red arrows) and an enlarged right intercostal artery (asterisk) (D: Th5 axial; E: Th6 axial). (F–H) MRI performed before admission demonstrated an intraspinal hematoma and intramedullary signal changes in the cervical and thoracic spinal cord (F: sagittal T2-weighted image; G: Th5 axial; H: Th6 axial). Abbreviations: CT, computed tomography; MRI, magnetic resonance imaging; ASSDH, acute spinal subdural hematoma

A whole-spine CT scan performed at the referring hospital immediately prior to transfer revealed an intraspinal hematoma extending from the epidural space of the clivus to the cervical and thoracic spinal canal (Figures 1A-1C). Repeat contrast-enhanced CT performed upon arrival demonstrated a focal contrast extravasation (“spot sign”) at the right T5-6 level and enlargement of the right intercostal artery (Figures 1D, 1E). Whole-spine MRI obtained before admission showed a subdural hematoma and intramedullary signal changes from the cervical to the thoracic spinal cord (Figures 1F-1H). At the T5-6 level, the spinal cord was markedly flattened with significant deformation, indicating severe compression.

**Figure 2:**
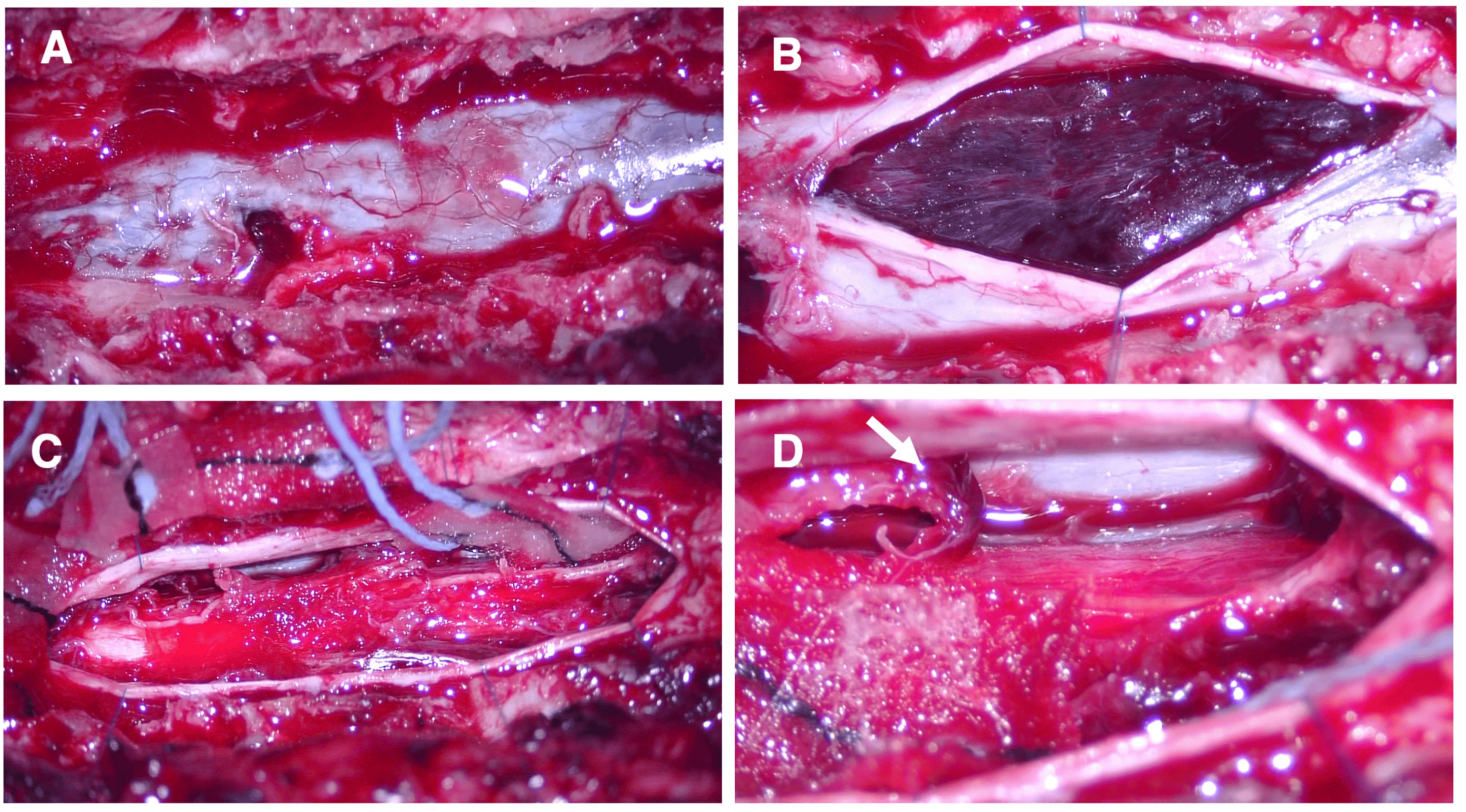
Intraoperative findings of acute spinal subdural hematoma (A) Findings after T4–7 laminectomy showed no epidural hematoma. (B) A massive subdural hematoma was found upon dural incision. (C) A photograph after hematoma removal. (D) A ruptured vascular wall (white arrow) was observed at the T5–6 level on the right side of the spinal cord. Abbreviations: ASSDH, acute spinal subdural hematoma

Because of rapid neurological deterioration, anticoagulation was immediately reversed with intravenous vitamin K and four-factor prothrombin complex concentrate. Emergency surgery was performed six hours after symptom onset. A T4-7 laminectomy revealed no epidural hematoma (Figure 2A). Opening the dura exposed a massive subdural hematoma (Figure 2B), which was evacuated (Figure 2C). A focal rupture of a radiculomedullary vessel corresponding to the CT “spot sign” was confirmed intraoperatively at the right T5-6 level (Figure 2D).

Despite intensive rehabilitation, no neurological improvement was observed at six months postoperatively, and the patient remained paraplegic (ASIA A).

## Discussion

Spontaneous spinal subdural hematoma is a rare clinical entity, and its pathophysiology remains incompletely understood [1-3]. Among previously reported cases, none have described the presence of a “spot sign” on contrast-enhanced CT, a radiological marker widely recognized in spontaneous intracerebral hemorrhage as an indicator of active bleeding [4]. In the present case, the spot sign corresponded intraoperatively to a focal rupture of a radiculomedullary artery, suggesting that the same concept may be applicable to spinal hematomas. This imaging feature may therefore provide a valuable clue for identifying the bleeding source in patients presenting with extensive spinal subdural hematomas, in whom the origin of hemorrhage is often unclear.

The mechanism of arterial rupture in this case is likely multifactorial. The patient had a markedly elevated PT-INR due to long-term warfarin therapy, which may have increased vessel fragility and impaired coagulation, predisposing the artery to rupture even in the absence of trauma. Additionally, sudden increases in intrathoracic or intra-abdominal pressure - such as straining - have been proposed as a potential trigger for abrupt shifts in intraspinal pressure, resulting in tearing of radicular or radiculomedullary vessels. The combination of anticoagulation and mechanical stress may therefore explain the abrupt onset observed in this patient.

A systematic review by Rettenmaier et al. demonstrated that spontaneous spinal subdural hematoma most frequently involves the thoracic spine and often results in poor neurological outcomes [5]. Our patient similarly exhibited extensive thoracic involvement and, unfortunately, showed no recovery despite early decompression. Although the hematoma had completely resolved on MRI at six months postoperatively, irreversible spinal cord injury was presumed due to prolonged severe compression, as indicated by significant intramedullary hyperintensity on preoperative MRI.

This case highlights several important clinical considerations. First, contrast-enhanced CT may play a greater role in the evaluation of acute spinal hematomas than previously recognized. The identification of a spot sign may indicate active bleeding and help localize the responsible vessel before surgery. Second, early correction of coagulopathy remains crucial, particularly in anticoagulated patients presenting with rapid neurological decline [6,7].

This report also has limitations. It is a single case, and angiographic evaluation was not performed before surgery due to the patient’s rapid deterioration, limiting our ability to confirm vascular abnormalities beyond intraoperative findings. Additionally, histopathological examination of the ruptured vessel was not feasible.

In summary, this case demonstrates that a CT spot sign, although well described in intracerebral hemorrhage, may also appear in spinal subdural hematomas and can correlate with active bleeding from a radiculomedullary artery. Further accumulation of similar cases is needed to clarify the diagnostic value and prognostic implications of this imaging finding.

## Conclusions

ASSDH is a rare but potentially devastating condition requiring rapid diagnosis and surgical decompression. In this case, a contrast-enhanced CT “spot sign” corresponded intraoperatively to an actively ruptured radiculomedullary artery, suggesting that this imaging feature - well known in intracerebral hemorrhage - may also be applicable to spinal hematomas. Recognition of a spinal spot sign may help identify active bleeding and guide timely intervention, particularly in anticoagulated patients presenting with abrupt neurological deterioration. Although this is a single case, the findings highlight the potential diagnostic value of contrast-enhanced CT in ASSDH, and further accumulation of cases is needed to clarify its clinical significance.
